# Insights into the Problem of Alarm Fatigue with Physiologic Monitor Devices: A Comprehensive Observational Study of Consecutive Intensive Care Unit Patients

**DOI:** 10.1371/journal.pone.0110274

**Published:** 2014-10-22

**Authors:** Barbara J. Drew, Patricia Harris, Jessica K. Zègre-Hemsey, Tina Mammone, Daniel Schindler, Rebeca Salas-Boni, Yong Bai, Adelita Tinoco, Quan Ding, Xiao Hu

**Affiliations:** 1 Department of Physiological Nursing, University of California San Francisco, San Francisco, California, United States of America; 2 School of Nursing, University of North Carolina, Chapel Hill, North Carolina, United States of America; 3 Department of Nursing, University of California San Francisco Medical Center, San Francisco, California, United States of America; Johns Hopkins University SOM, United States of America

## Abstract

**Purpose:**

Physiologic monitors are plagued with alarms that create a cacophony of sounds and visual alerts causing “alarm fatigue” which creates an unsafe patient environment because a life-threatening event may be missed in this milieu of sensory overload. Using a state-of-the-art technology acquisition infrastructure, all monitor data including 7 ECG leads, all pressure, SpO2, and respiration waveforms as well as user settings and alarms were stored on 461 adults treated in intensive care units. Using a well-defined alarm annotation protocol, nurse scientists with 95% inter-rater reliability annotated 12,671 arrhythmia alarms.

**Results:**

A total of 2,558,760 unique alarms occurred in the 31-day study period: arrhythmia, 1,154,201; parameter, 612,927; technical, 791,632. There were 381,560 audible alarms for an audible alarm burden of 187/bed/day. 88.8% of the 12,671 annotated arrhythmia alarms were false positives. Conditions causing excessive alarms included inappropriate alarm settings, persistent atrial fibrillation, and non-actionable events such as PVC's and brief spikes in ST segments. Low amplitude QRS complexes in some, but not all available ECG leads caused undercounting and false arrhythmia alarms. Wide QRS complexes due to bundle branch block or ventricular pacemaker rhythm caused false alarms. 93% of the 168 true ventricular tachycardia alarms were not sustained long enough to warrant treatment.

**Discussion:**

The excessive number of physiologic monitor alarms is a complex interplay of inappropriate user settings, patient conditions, and algorithm deficiencies. Device solutions should focus on use of all available ECG leads to identify non-artifact leads and leads with adequate QRS amplitude. Devices should provide prompts to aide in more appropriate tailoring of alarm settings to individual patients. Atrial fibrillation alarms should be limited to new onset and termination of the arrhythmia and delays for ST-segment and other parameter alarms should be configurable. Because computer devices are more reliable than humans, an opportunity exists to improve physiologic monitoring and reduce alarm fatigue.

## Introduction

**Figure 1:**
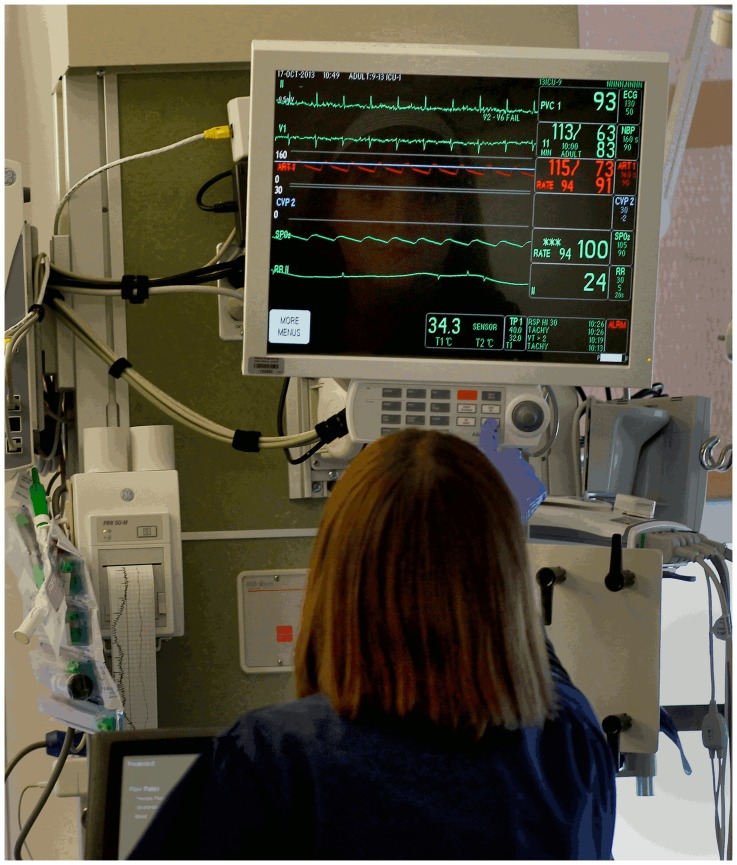
Physiologic monitor device in Intensive Care Unit. Bedside patient monitor (GE Healthcare, Milwaukee, WI) displays multiple physiologic waveforms and vital sign measurements. The nurse pictured here gave written informed consent to publish this photograph supplied by the San Francisco Chronicle newspaper (with permission) for their story on alarm fatigue at: http://www.sfgate.com/health/article/Hospitals-look-to-reduce-danger-of-alarm-fatigue-4918018.php.

Critical care clinicians rely heavily upon information provided by physiologic monitor devices for minute-to-minute clinical decision-making in hospital intensive care units (ICUs) (Figure 1). Waveforms routinely displayed at the bedside and central stations include electrocardiograms (ECGs), respiration, invasive pressures (arterial, pulmonary artery, central venous, intra-cranial), and peripheral oxygen saturation (SpO_2_). The monitor device’s computer also makes frequent measurements of a myriad of vital sign parameters such as heart rate, respiratory rate, SpO_2_, systolic, diastolic, and mean values for all available pressures, just to name a few. When any of these individual parameters fall outside the “too low” or “too high” alarm thresholds for a few seconds, an alarm is triggered which may sound an audible tone or visual text message.

In addition to the plethora of parameter alarms, physiologic monitor devices also contain arrhythmia computer algorithms that trigger an alarm when a change in cardiac rhythm is detected. Critical arrhythmia alarms such as asystole or ventricular fibrillation are configured as “latching” alarms that produce incessant sounds that do not cease until a clinician silences the alarm. All too often, these incessant alarms are triggered by something as benign as motion artifact from activities such as brushing one’s teeth. The end result is that clinicians are exposed to a high number of physiologic monitor alarms over the span of their 8–12 hour shift causing excessive alarm burden.

In 2010, excessive alarm burden was exposed by the press as a patient safety concern by the highly-publicized death of a patient who was being monitored at a prestigious medical center. Despite multiple low heart rate alarms that occurred prior to the patient’s cardiac arrest, no-one working on the unit that day recalled hearing the alarms. In the investigation that ensued, the Centers for Medicare and Medicaid Services reported: “Nurses not recalling hearing low heart rate alarms were indicative of *alarm fatigue* which contributed to the patient’s death” [1].

Alarm fatigue occurs when clinicians are desensitized by numerous alarms, many of which are false or clinically irrelevant. As a result, the cacophony of alarm sounds becomes “background noise” that is perceived as the normal working environment in the ICU. Importantly, alarms may be silenced at the central station without checking the patient or permanently disabled by clinicians who find the constant audible or textual messages bothersome. The Association for the Advancement of Medical Instrumentation and the U.S. Food & Drug Administration have warned of deaths due to alarm silencing on patient monitor devices [2]. Likewise, a number of other federal agencies and national organizations have issued alerts about alarm fatigue being a major patient safety concern. For example, the Emergency Care Research Institute, a leading nonprofit organization, lists alarm fatigue as the number one health technology hazard for 2014 [3]. In addition, the Joint Commission that approves hospitals for accreditation issued an alarm safety alert in 2013; in 2014, they established alarm safety as a National Patient Safety Goal, and further regulations will be compulsory in 2016 [4].

To date, there has not been a comprehensive investigation of the frequency, types, and accuracy of physiologic monitor alarms collected in a “real-world” ICU setting. For this reason, nurse and engineer scientists in the ECG Monitoring Research Laboratory at the University of California, San Francisco (UCSF) designed a study to provide complete data on monitor alarms. In addition to alarm frequency and accuracy, further questions the investigators explored were: 1) Are false arrhythmia alarms due to poor ECG signal quality that might be resolved by a better skin prep/electrode regimen? 2) How important is it to analyze all available ECG leads for arrhythmia diagnosis? 3) How often are non-ECG waveforms (e.g., pressures, SpO2) needed for arrhythmia diagnosis? 4) How often are ventricular arrhythmia alarms clinically relevant in terms of meeting published practice guideline criteria for treatment in hospital settings?

The purpose of this paper is to report results of an initial analysis of data collected during the 31 days of March, 2013. We have included ECG figures of alarm conditions that illustrate all the key findings. Also discussed are insights that shed light on the problem of excessive alarm burden with recommendations to provide guidance for developing solutions to address the problem of clinical alarm fatigue.

## Methods

### Research Design and Setting

The UCSF Alarm Study used a prospective data collection design with a state-of-the-art technology infrastructure to collect all available physiologic waveforms, computer vital sign measurements, clinician alarm settings, and alarms that occurred in the medical center’s five adult ICUs. The patient populations treated in these five units span the breadth of clinical disorders (medical, surgical, cardiac, and neurologic) treated in a large tertiary-quaternary medical center as summarized in Table 1. The UCSF Committee on Human Research approved the study with waiver of patient consent because all ICU patients have physiologic monitoring as part of their routine care and acquisition and storage of this data did not influence their clinical care. A major advantage of the waiver of patient consent is that all consecutive patients treated in these ICUs were included in the study; no patients were excluded from the analysis. The nurse pictured in Figure 1 has provided consent for publication.

**Table 1:** UCSF Alarm Study Units.

Hospital Unit	# Beds	Patient Population
2 Intensive Care Units	32	Critically-ill adults with complex medical disorders and post-operative general surgery, solid organ transplant, acute kidney injury, acute and end-stage liver failure, sepsis, multiple organ dysfunction syndrome, and liver, pancreas or small bowel transplantation. Patients commonly on mechanical ventilation.
Cardiac Critical Care	16	Critically-ill adults with cardiac disorders, including cardiac medicine, cardiothoracic surgery, transplant (heart, lung, heart & lung), thoracic or vascular surgery. Patients with left ventricular assist devices, pacemakers, & implantable cardioverter devices.
2 Neuroscience Care Units	29	Critically-ill adults with neurological impairment (subarachnoid hemorrhage, stroke, brain tumors, traumatic brain injury) who undergo complex surgical and interventional procedures and patients going through the organ donation process.
**TOTAL:**	**77**	

### Collection of Waveform and Alarm Data

**Figure 2:**
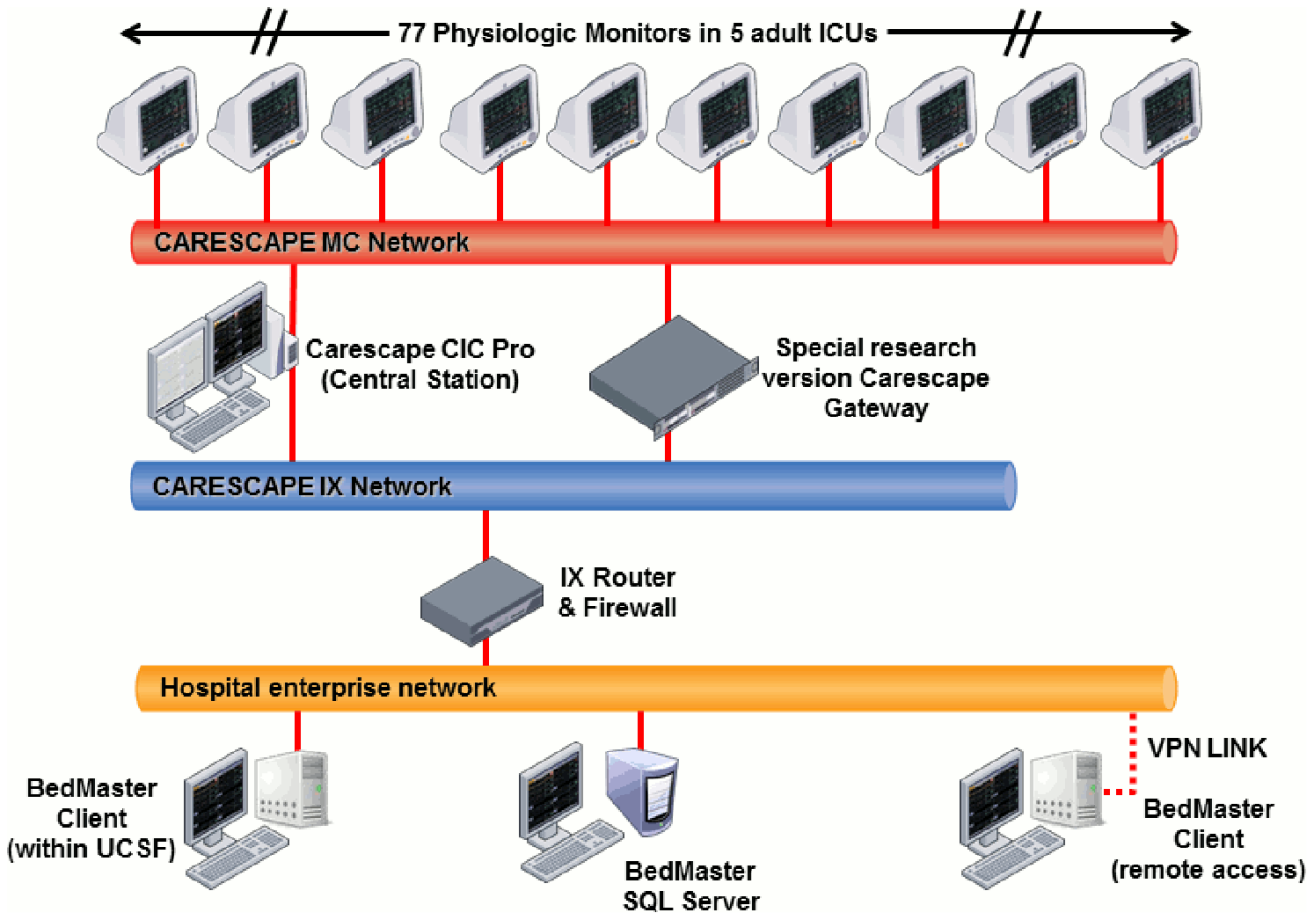
Hospital infrastructure to automatically store all physiologic monitor waveform and alarm data.

Figure 2 illustrates the hospital infrastructure that was installed to automatically store the physiologic monitor data for the UCSF Alarm Study. Each of the 77 ICU beds is equipped with a Solar 8000i bedside monitor (version 5.4 software, GE Healthcare, Milwaukee, WI) that acquires, processes, and stores data. A closed network connects all bedside monitors and central monitoring stations. The CARESCAPE Gateway system (GE Healthcare, Milwaukee, WI) enables study data to securely pass out of the network to an external server to be analyzed retrospectively.

A special research version of CARESCAPE Gateway was built to collect comprehensive alarm data such as nurse-determined individual patient alarm settings and all unique alarms including message (inaudible) and technical alarms. In prior research, investigators have reported only on audible alarms because they are thought to contribute more to alarm fatigue than inaudible alarms. Our rationale for collecting inaudible as well as audible alarms is that nurses (and sometimes patients) notice text message alarms displayed on the bedside monitor. Thus, they do raise concern about whether something is wrong and require thought about whether a response is necessary. Inaudible technical alarms may signal a problem that, if uncorrected, will lead to complete suspension of arrhythmia detection. Moreover, these technical alarms may provide a sensitive marker to determine the efficacy of interventions such as optimal electrode regimens. For example, a technical message on the monitor that says “Artifact” indicates a noisy ECG signal but it does not sound an audible alarm nor does it suspend arrhythmia detection. However, if the artifact continues, it will trigger an “Arrhythmia Suspend” alarm that completely suspends all (including lethal) arrhythmia detection, putting the patient in a unsafe environment.

BedMasterEx software (Excel Medical Electronics, Inc, Jupiter, FL) was installed to store physiologic waveforms, vital signs (device-measured parameters), alarm settings, and monitor alarms in a relational database (SQL Server™). The waveform data were saved in flat files following a proprietary format. The vendor of BedMasterEx software provided a command-line software utility to extract waveform data into Extensible Markup Langue (XML) files. The investigators further developed an application to parse these XML files, detect gaps in the data streams, alternations of signal channel configurations, and then assemble the waveform data into multiple binary files following the publicly available format from AD Instrument (Dunedin, New Zealand). These binary files can be reviewed using a free software application LabChart Reader from AD Instrument. In addition, these files can be readily loaded into analytics programs including Matlab for further analysis.

All waveform signals were acquired including ECG (240 Hz), invasive pressures (120 Hz), and SpO_2_ (60 Hz). Each waveform sample was represented with 12 bits. In addition, the scale factors of each channel as specified by BedMasterEx were stored in the corresponding fields in binary file header to enable the reconstruction of samples in the original physical units.

In the pilot phase of the study, the investigators realized they could not depend on the accuracy of the medical record numbers as inputted in the patient monitors to find monitor data for a given patient because of human errors at the bedside. To solve this problem, patient bed transfer histories were extracted from the hospital electronic medical record system. This established a correct association between medical record number and waveform data by determining the location of a given patient first and then retrieving the corresponding database records in the relational database based on the patient location and time information.

### Patient Monitoring ECG Lead Configuration and Alarm Default Settings

**Figure 3:**
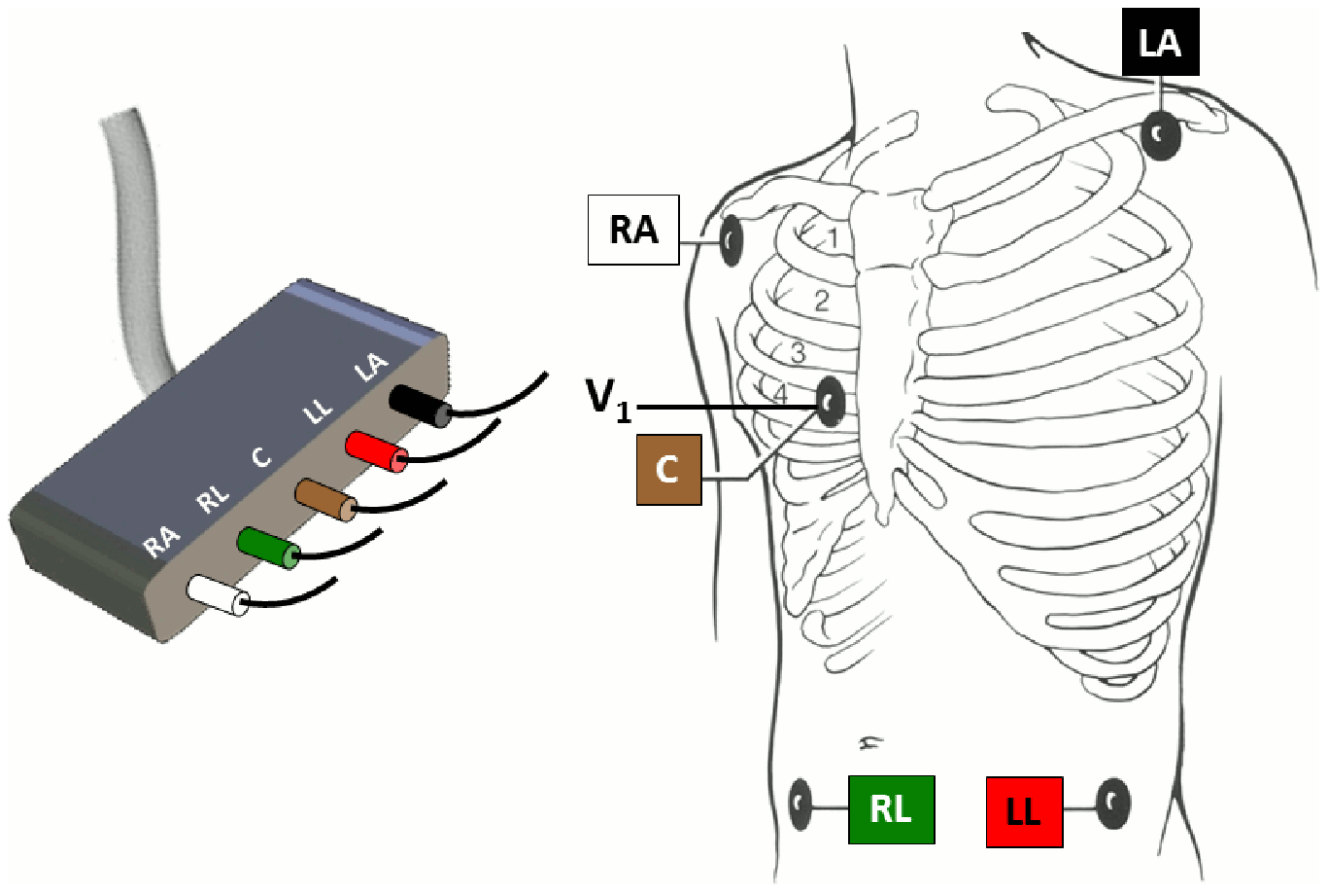
Patient monitoring ECG lead configuration. A 5-electrode lead configuration was used in all study ICUs with Mason-Likar electrode placement of the limb leads on the torso and one chest electrode that is routinely placed in the V_1_ location.

A five-electrode ECG lead configuration was used for all patients (Figure 3) resulting in seven available leads (I, II, III, aVR, aVL, aVF, and V). Although only two ECG leads were displayed on the bedside monitor, all seven leads were stored and available to the investigators for arrhythmia alarm annotation.

Table 2 shows the alarm default settings recommended by the manufacturer (factory defaults) and the UCSF default settings used during the 31-day study period. Some non-lethal alarms can be configured by the nurse to change an audible alarm to an inaudible message. However, all technical alarms are not configurable so the factory default settings are permanent.

**Table 2:** Alarm Default Settings for Adult ICUs during the Study Period.

Patient Status Arrhythmia Alarms		
Alarm Sounds: Crisis, 3 beeps continually; Warning, 2 beeps; Advisory, 1 beep; Message, inaudible text		
Alarm	Factory Default	UCSF Default
Asystole	Crisis	Crisis
Ventricular Fibrillation/Ventricular Tachycardia	Crisis	Crisis
Ventricular Tachycardia	Crisis	Crisis
Ventricular Tachycardia >2	Crisis	Advisory
Ventricular Bradycardia	Crisis	Warning
Accelerated Ventricular Rhythm	Message	Warning
Pause	Message	Warning
Tachycardia	Message	Advisory
Bradycardia	Message	Advisory
R on T	Message	Message
Couplet	Message	Message
Bigeminy	Message	Message
Trigeminy	Message	Message
Premature ventricular contraction (PVC)	Message	Message
Irregular	Message	Message
Atrial Fibrillation	Message	Advisory
**Patient Status Parameter Limit Violation Alarms (selected)**		
**Alarm Sounds: Warning, 2 beeps; Advisory, 1 beep; Message, inaudible text**		
**Alarm**	**Factory Default**	**UCSF Default**
Heart Rate	50/150 Warning	50/130 Warning
PVC/minute	6 Message	10 Message
Invasive arterial pressure	Advisory	90/160 Warning
Noninvasive blood pressure	Advisory	90/160 Advisory
ST segment	Advisory	Advisory
Respiratory Rate	Message	Warning
No Breath/Apnea	Warning	Warning
Peripheral oxygen saturation (SpO_2_)	Advisory	90% Advisory
**System Status Technical Alarms (selected)**		
**Alarm Sounds: Warning, continuous foghorn tone; Message, inaudible text**		
**Alarm**	**Factory Default**	**UCSF Default**
Artifact	Message	Message
Lead Fail (single lead I or II or III or RL or V)	Message	Message
ECG Leads Fail	Warning	Warning
Respiratory Leads Fail	Warning	Warning
Arrhythmia Suspend	Warning	Warning
Invasive Pressure Sensor Fail	Warning	Warning
Noninvasive Blood Pressure Deflation Failure	Warning	Warning
Noninvasive Blood Pressure Exceed 3 Minutes	Warning	Warning
Noninvasive Blood Pressure Excessive Pressure 200	Warning	Warning
Noninvasive Blood Pressure Invalid Command	Warning	Warning

### Annotation of Arrhythmia Alarms

Six arrhythmia alarms that were considered clinically important enough to be set as audible alarms were determined to be true or false by clinical experts using a standardized protocol developed for the study. The alarm annotation protocol is shown in Table 3 with alarm definitions and the criteria used for judging whether the six alarms were true or false positives. The annotators were four nurse scientists (co-authors PH, JZH, TM, DS), all of whom had PhD training and clinical experience with physiologic monitoring devices in hospital settings. All annotators completed a formal 10-week course in clinical electrocardiography that has been taught by the Principal Investigator (PI, BJD) at UCSF for the past 33 years. In addition, they underwent a 3-hour alarm annotation certification course taught by the PI that was video-taped for review as needed. The PI and four annotators met in-person weekly and were in almost daily email communication during the alarm annotation period to review cases and reach consensus about an accurate and consistent method of arrhythmia alarm interpretation.

**Table 3:** Alarm Annotation Protocol.

Alarm Label & Algorithm Definition	Proof of True versus False Alarm by Investigator
**1. ASYSTOLE** Displayed heart rate drops to zero. No QRS detected for ∼5–6 seconds	**Proof of True Positive:** (either #1 or #2 confirms true alarm)
	1. Simultaneous drop in invasive arterial or pulmonary artery (PA) pressure to near zero
	2. Documentation from electronic medical record (EMR) of asystolic cardiac arrest at same time
	**Proof of False Positive:** (any of the following confirms false positive alarm)
	1. No simultaneous decrease in invasive arterial or PA pressure
	2. A visible QRS is evident in at least one ECG lead (examine all 7 available leads)
	3. Good quality SpO2 signal has pulsatile waveform that matches rate of underlying baseline rhythm
	4. ASYSTOLE alarm duration is >60 seconds but there is no EMR documentation that it was recognized clinically (syncope, seizure, loss of consciousness, cardiac arrest)
**2. VFIB/VTAC** Coarse flutter waves without QRS complexes	**Proof of True Positive:** (either #1 or #2 confirms true alarm)
	1. Simultaneous drop in invasive arterial or PA pressure to near zero
	2. Documentation from EMR of ventricular tachycardia or fibrillation cardiac arrest at same time
	**Proof of False Positive:** (any of the following confirms false positive alarm)
	1. No simultaneous decrease in invasive arterial or PA pressure
	2. There are QRS complexes with the same R–R intervals as the patient’s baseline rhythm evident in any ECG lead throughout the alarm event
	3. Good quality SpO_2_ signal has pulsatile waveform that matches rate of underlying baseline rhythm
	4. VFIB/VTAC alarm duration is >60 seconds but there is no EMR documentation that it was recognized clinically (syncope, seizure, loss of consciousness, cardiac arrest)
**3. ACC VENT** ≥6 ventricular beats with HR 50–100 bpm	**Proof of True Positive:**
	1. Wide QRS beats are not preceded by a P wave with a consistent PR interval
	2. Fusion beats are evident at the transition between ventricular rhythm and sinus rhythm
	**Proof of False Positive:** (either #1 or #2 confirms false positive alarm)
	1. Event is sinus rhythm with BBB (P waves prior to each wide beat with consistent PR interval)
	2. Patient is known to have ventricular pacemaker; event QRS matches paced rhythm on standard “diagnostic” 12-lead ECG
**4. VTACH** ≥6 consecutive PVCs with rate ≥100 bpm	**Proof of True Positive:** (any of the following confirms true positive alarm)
	1. Simultaneous drop in invasive arterial or PA pressure
	2. Documentation from EMR of VT at same time; standard 12-lead ECG documentation of VT read by cardiologist
	3. Atrioventricular (AV) dissociation is evident throughout the wide QRS tachycardia in any ECG lead
	4. Event wide QRS morphology is different than patient’s baseline rhythm with BBB
	**Proof of False Positive:** (any of the following confirms false positive alarm)
	1. No simultaneous change in invasive arterial or PA pressure (if it is “slow” VT with rate 100–150, there will be less decrease in pressure waveform amplitude)
	2. There are QRS complexes with the same R–R intervals as the patient’s baseline rhythm evident in any ECG lead throughout the alarm event
	3. Good quality SpO_2_ signal has pulsatile waveform that matches rate of underlying baseline rhythm
	4. VTACH alarm duration is >60 seconds but there is no EMR documentation that it was recognized clinically (syncope, seizure, loss of consciousness, cardiac arrest)
	5. Event has the same wide QRS complex morphology in all 7 ECG leads as the patient’s baseline rhythm with right or left BBB; additional confirmation if sinus P waves are evident prior to each QRS or the rhythm has no discernable P waves but is randomly irregular indicating atrial fibrillation
	6. Event is due to intermittent ventricular pacing (visible pacer spikes before each wide QRS or QRS in all 7 leads matches a standard “diagnostic” 12-lead ECG acquired during ventricular pacing)
**5. PAUSE** 3-second interval without a QRS complex	**Proof of True Positive:** (either #1 or #2 confirms true alarm)
	1. Simultaneous pause on invasive arterial or PA waveform
	2. Simultaneous pause on good quality SpO_2_ waveform
	**Proof of False Positive:** (any of the following confirms false positive alarm)
	1. No simultaneous pause in invasive arterial or PA pressure
	2. No simultaneous pause on good quality SpO_2_ waveform
	3. There is a visible QRS during the pause (may be low amplitude) in any of the 7 available leads
**6. VBRADY** ≥3 consecutive ventricular beats with HR ≤50 bpm	**Proof of True Positive:**
	1. Rhythm is complete heart block with ventricular escape rhythm
	2. Rhythm is sinus node arrest with ventricular escape rhythm
	**Proof of False Positive:** (either #1 or #2 confirms false positive alarm)
	1. Event is sinus bradycardia with BBB (P waves prior to each beat with consistent PR interval)
	2. Patient is known to have pacemaker; event QRS in all 7 leads matches paced rhythm on “diagnostic” 12-lead ECG

**Figure 4:**
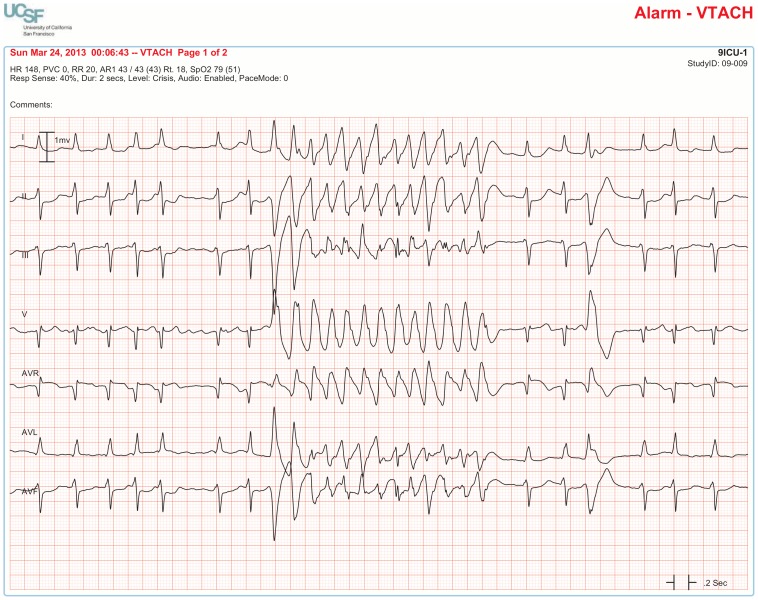
True positive ventricular tachycardia alarm using seven available ECG leads for diagnosis. Page one of the alarm annotation analysis tool shows a 10-second rhythm strip of all seven available ECG leads at the time that a ventricular tachycardia alarm was triggered. In this and subsequent Figures, ECG Leads are displayed from top to bottom in the following sequence: Lead I, II, III, V (typically V_1_), aVR, aVL, aVF. As evident at the beginning of the rhythm strip, the patient has an underlying rhythm of atrial fibrillation with a rapid ventricular rate of about 140. There is an isolated ventricular premature beat (4^th^ beat from the end) and its QRS morphology is identical to the initial beat of the alarm event. Knowing that the event is initiated by a ventricular ectopic beat provides strong evidence that this event is a true ventricular tachycardia alarm.

**Figure 5:**
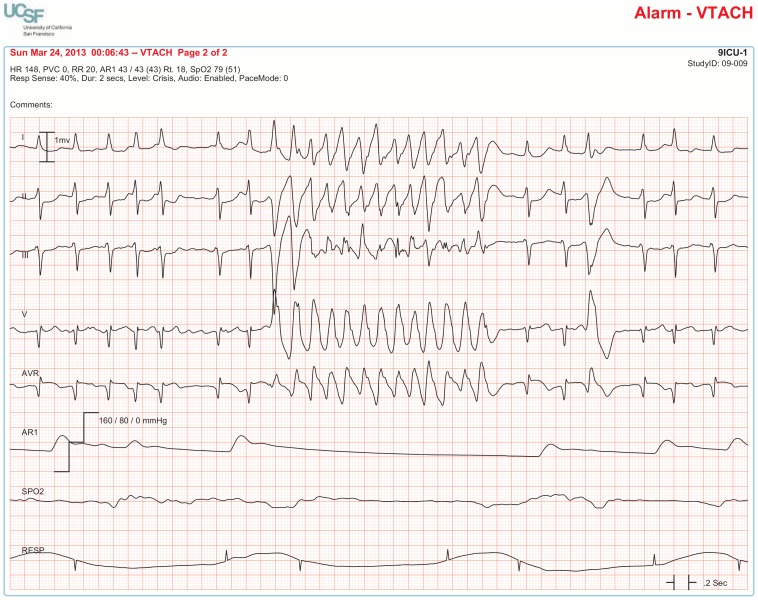
True positive ventricular tachycardia alarm using non-ECG waveforms for diagnosis. Page 2 of the alarm annotation analysis tool depicts the same alarm event as in Figure 4 with all available non-ECG waveforms. Additional proof that this is a true ventricular tachycardia alarm is provided by observing cessation of the arterial blood pressure waveform that falls to near zero during the arrhythmia.

**Figure 6:**
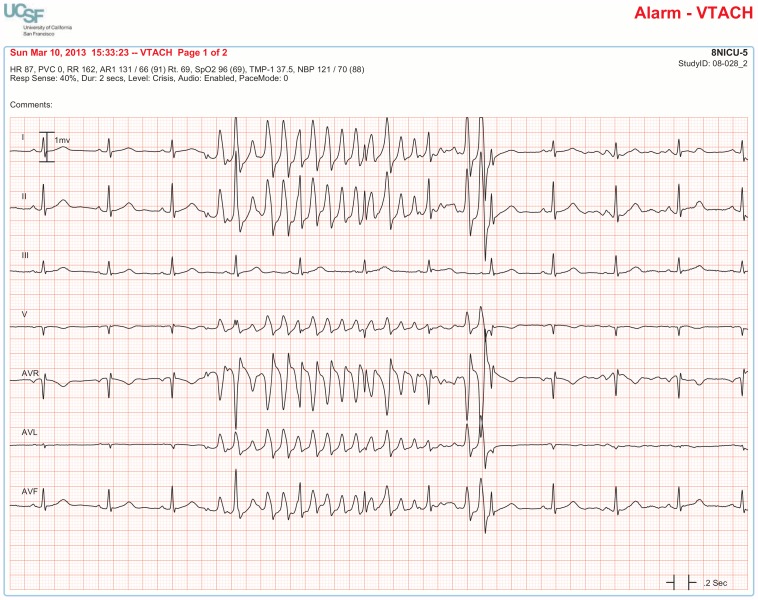
False positive ventricular tachycardia alarm using seven available ECG leads for diagnosis. Page one of the alarm annotation analysis tool in a second patient with a ventricular tachycardia alarm. Proof that this is a false positive alarm is provided by observing Lead III that shows clearly-visible P-QRS-T waveforms indicating normal sinus rhythm. All six remaining ECG leads show artifact that mimics ventricular tachycardia. It is important to point out that Lead III is not one of the two leads routinely displayed on the bedside monitor in our ICUs so unless all available leads are reviewed, a misdiagnosis would be made of rapid polymorphic ventricular tachycardia. This type of rapid, repetitive artifact on the ECG is often created during patient monitoring by motion artifact during activities of daily living. The non-artifact lead (Lead III) uses the left arm and left leg electrodes but not the right arm electrode. So, this is likely to be a right-handed patient doing something like brushing teeth.

**Figure 7:**
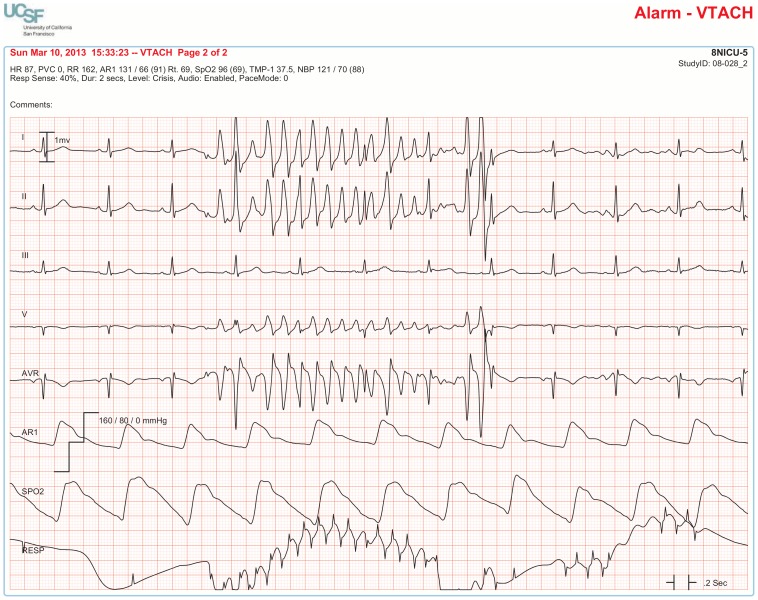
False positive ventricular tachycardia alarm using non-ECG waveforms for diagnosis. Page 2 of the alarm annotation analysis tool depicts the same alarm event as in Figure 6 showing all available non-ECG waveforms. Additional proof that this is a false ventricular tachycardia alarm is provided by the following: a.) no change in the arterial pressure waveform during the event, b.) arterial waveform pulsations match the normal sinus rhythm rate, and c.) SpO_2_ waveform pulsations match the normal sinus rhythm rate. Of interest, the same artifact that contaminates the ECG signal also contaminates the respiratory waveform, as evidenced by an erroneous device-measured respiratory rate of 162 breaths per minute.

The BedMasterEx vendor provided two pages of waveforms for each alarm that were used by the annotators in their analysis. The first page displayed a 10-second rhythm strip of the seven available ECG leads at the time the alarm was triggered. The second page displayed the same alarm event with fewer ECG leads and all available non-ECG waveforms. When more than a 10-second rhythm strip was necessary for alarm annotation, the annotator pulled up the alarm on the BedMaster Client viewer and scrolled backward and forward as needed. An example of the first and second page of a true positive ventricular tachycardia alarm is shown in Figures 4 and 5. Figures 6 and 7 illustrate an example of a false positive ventricular tachycardia alarm.

Inter-rater reliability of alarm annotation was tested by randomly selecting 300 alarms that were rated twice by pairs of the annotators. A Cohen’s Kappa was run to compare “Rater 1″ to “Rater 2.” There was 95% agreement as to whether the alarm was a true or false positive (Cohen’s Kappa score of 0.86). The disagreements were settled by the PI and the database was corrected accordingly.

### Analysis of Alarm Frequency and Type

All unique alarms for the 31-day period were grouped into three main categories: arrhythmia, parameter, and technical alarms. For analysis and reporting purposes, individual alarms of similar type were grouped into 17 categories as shown in Table 4.

**Table 4:** Schema for Counting and Reporting Physiologic Monitor Device Alarms.

Arrhythmia Alarms			
Alarm Condition	Short Label	Definition	
1. Accelerated Ventricular Rhythm	Acc Vent	≥6 ventricular beats with heart rate between 50-100	
2. Atrial Fibrillation	Afib	Irregular timing of QRS complexes and absenceof preceding P waves	
3. Asystole	Asystole	Heart rate drops to zero; typically no QRS for 5–6 seconds	
4. Pause	Pause	No QRS for a 3-second interval	
5. Ventricular Bradycardia	V Brady	≥3 consecutive ventricular beats at an average rate ≤50	
6. Ventricular Fibrillation	Vfib/Vtac	Course flutter waves without QRS complexes	
7. Ventricular Tachycardia	Vtach	≥6 consecutive ventricular beats at rate ≥100	
8. Premature VentricularContractions	All PVC	VT>2	3-5 consecutive ventricular beats at rate ≥100
		PVC	Isolated PVCs
		R on T	PVC falls on the ST or T wave portionof previous beat
		Couplet	Two consecutive PVCs with rate >100
		Bigeminy	PVC alternates with a non-ventricular beat for ≥3 cycles
		Trigeminy	PVC alternates with 2 non-ventricular beats for ≥3 cycles
		PVC = > X	PVC count is equal to or>than user-defined limit
**Parameter Alarms (“Too Low – Too High” Vital Sign Measurements)**			
9. Heart Rate	All HR	HR<or>user-defined limit determined from ECG waveform (HR)	
		HR<or>user-defined limit determined from SpO2 waveform (SpO2 Rate)	
		HR<or>user-defined limit determined from arterial pressurewaveform (ART Rate or FEM Rate)	
		Bradycardia arrhythmia alarm: 8 R–R intervals fall below user-definedlow HR limit setting	
		Tachycardia arrhythmia alarm: 8 R–R intervals occur aboveuser-defined high HR limit setting	
10. Respiratory Rate	All RR	Respiratory rate<or>user-defined limit	
		No breaths detected for user-defined period of seconds (Apnea Alarm)	
11. Oxygen Saturation	All SpO_2_	SpO_2_< or>user-defined limit determined from pulse oximetry sensor	
12. Invasive Arterial Pressure	All ART	Systolic = or<or>user-defined limit	
		Diastolic = or<or>user-defined limit	
		Mean = or<or>user-defined limit	
13. Noninvasive Blood Pressure	All NIBP	Systolic = or<or>user-defined limit	
		Diastolic = or<or>user-defined limit	
		Mean = or<or>user-defined limit	
14. Central & Intra-cardiac Pressure with invasive hemodynamic monitoring	All Heart Pressures	Systolic = or<or>user-defined limit for CVP, RAP, PAP, LAP	
		Diastolic = or<or>user-defined limit for CVP, RAP, PAP, LAP	
		Mean = or<or>user-defined limit for CVP, RAP, PAP, LAP	
15. Intra-cranial Pressure	All ICP	ICP mean = or<or>user-defined limit	
16. ST-segment Amplitude	All ST	Lead I or II or III or aVR or aVL or aVF or V1 or V2 or V3 or V4 or V5 or V6 ST<or>than PR segment amplitude by user-definedsetting (hospital default, ±2 millimeters)	
**Technical Alarms**			
17. Problem with artifact, sensors,probes, line disconnects, etc.	All Technical	Artifact: noisy signal on ECG	
		Arrhythmia suspend: no arrhythmia detection due to sustained artifact	
		Arrhythmia Off	
		ECG Leads Fail or No ECG or individual Lead Fail (I, II, III, RL, V)	
		Respiratory Lead Fail	
		Sensor Fail for ART or FEM or ICP or CVP or RAP or PAP or LAP	
		SpO_2_ :Probe Off or Probe Fail or Low Signal or IncompatibleCable or Connect Probe	
		Noninvasive BP: invalid command or ExcessivePressure 200 or Exceeded 3 min or Deflation Failure orInflation Time Exceeded	
		Line Disconnect for PA or ART or FEM or CVP	

CVP = central venous pressure; RAP = right atrial pressure; PAP = pulmonary artery pressure; LAP = left atrial pressure; ART = invasive arterial line; FEM = invasive arterial line in femoral site.

### Analysis of ECG Signal Quality

Each annotated arrhythmia alarm was rated by the investigators as having good, fair, or poor signal quality. Good signal quality was defined as a clearly visible P-QRS-T waveform across all available leads with little to no noise, baseline wander, or leads off. Fair signal quality was defined as moderate noise or baseline wander but having identifiable QRS complexes for basic rhythm/rate detection. Poor signal quality was defined as being unanalyzable because of excessive noise, baseline wander or leads off.

### Patient Data

Data were collected on patient demographics (age, race, ethnicity, gender), final diagnosis, and patient outcome variables of interest including cardiac or respiratory arrest (“Code Blue”) and in-hospital death.

Electrocardiographic conditions hypothesized to trigger false ventricular arrhythmia alarms were collected from hospital-acquired standard “diagnostic” 12-lead ECGs including the presence of a widened QRS complex due to right or left bundle branch block or ventricular pacemaker rhythms. In annotating alarms for patients with pacemakers, data were collected on whether or not nurses had activated the PaceMode feature on the monitor device that provides a separate algorithm with higher sampling rate to detect pacemaker stimuli.

Patient factors hypothesized to cause alarms by affecting signal quality or QRS amplitude were also collected including body mass index, current smoker status that is likely to cause agitation with nicotine withdrawal, confused mental status, tremor, seizures, and use of devices known to cause electrical interference such as hypothermia or ventricular assist devices.

### Statistical Analyses

Descriptive statistics (frequencies, proportions, means and standard deviations) were used to depict the total number of unique alarms and alarms by category. Audible alarm burden was calculated as the number of audible alarms per bed per day. Hourly alarm rates for each patient were determined by first calculating each patient’s total monitoring time. A computer algorithm developed by one of the investigators (YB) eliminated periods when the patient was detached from the ICU monitor device (e.g., for surgery, cardiac catheterization, or other off-unit diagnostic procedure), as evidenced by flat lines on the seven ECG leads. Hourly rates for any alarm could then be calculated by dividing the number of alarms by the patient’s total monitoring time. By normalizing the alarms in this way, patients with different ICU lengths of stay could be compared and an analysis could be made of patient factors associated with a high hourly rate of alarms.

## Results

### Sample

A total of 461 patients were treated in the five ICUs during the 31-day period of March, 2013 and all were included in the analysis. The average daily census during this month for the 77 ICU beds was 65.9 patients. Patients’ average monitoring time was 104.5 hours; median, 52.9 hours; minimum to maximum range, 5.3–744.0 hours. Of the total of 461 subjects, 17 (3.7%) experienced a cardiac or respiratory arrest during the 31-day period of the study. Overall in-hospital mortality for the cohort of 461 subjects was 35 (7.6%).

The sample had a mean age of 60±17 years and 250 (54%) were male. Race/ethnicity reflected the diversity of the San Francisco Bay area with 180 (39%) from non-white minorities. Of the total 461 patients, 83 (18%) were treated for a cardiac medical or surgical diagnosis, 197 (43%) were treated for a neurologic or neurosurgical diagnosis, and 181 (39%) were treated for another medical-surgical (pulmonary, sepsis, multi-system organ failure, etc) diagnosis. One hundred sixty-five patients (35.8%) were on mechanical ventilation. Patients who had a baseline cardiac rhythm with a wide QRS complex were as follows: right or left bundle branch block, 41 (8.9%); temporary or permanent ventricular pacemaker rhythm, 48 (10.4%).

### Overall Alarm Frequency and Type

Over the total monitoring time of 48,173 hours, there were a total of 2,558,760 unique audible and inaudible (visual text message) alarms in the five ICUs during the 31-day period. Of this total, 1,154,201 were arrhythmia alarms, 612,927 were vital sign parameter (“too low - too high”) alarms, and 791,632 were technical alarms. UCSF’s default settings restrict the number of alarms that generate an audible tone to just those that are considered clinically important. Despite this restriction, there were a total of 381,560 audible alarms, for an average alarm burden of 187 audible alarms per bed per day.

### Most Frequently-Occurring Alarms

**Figure 8:**
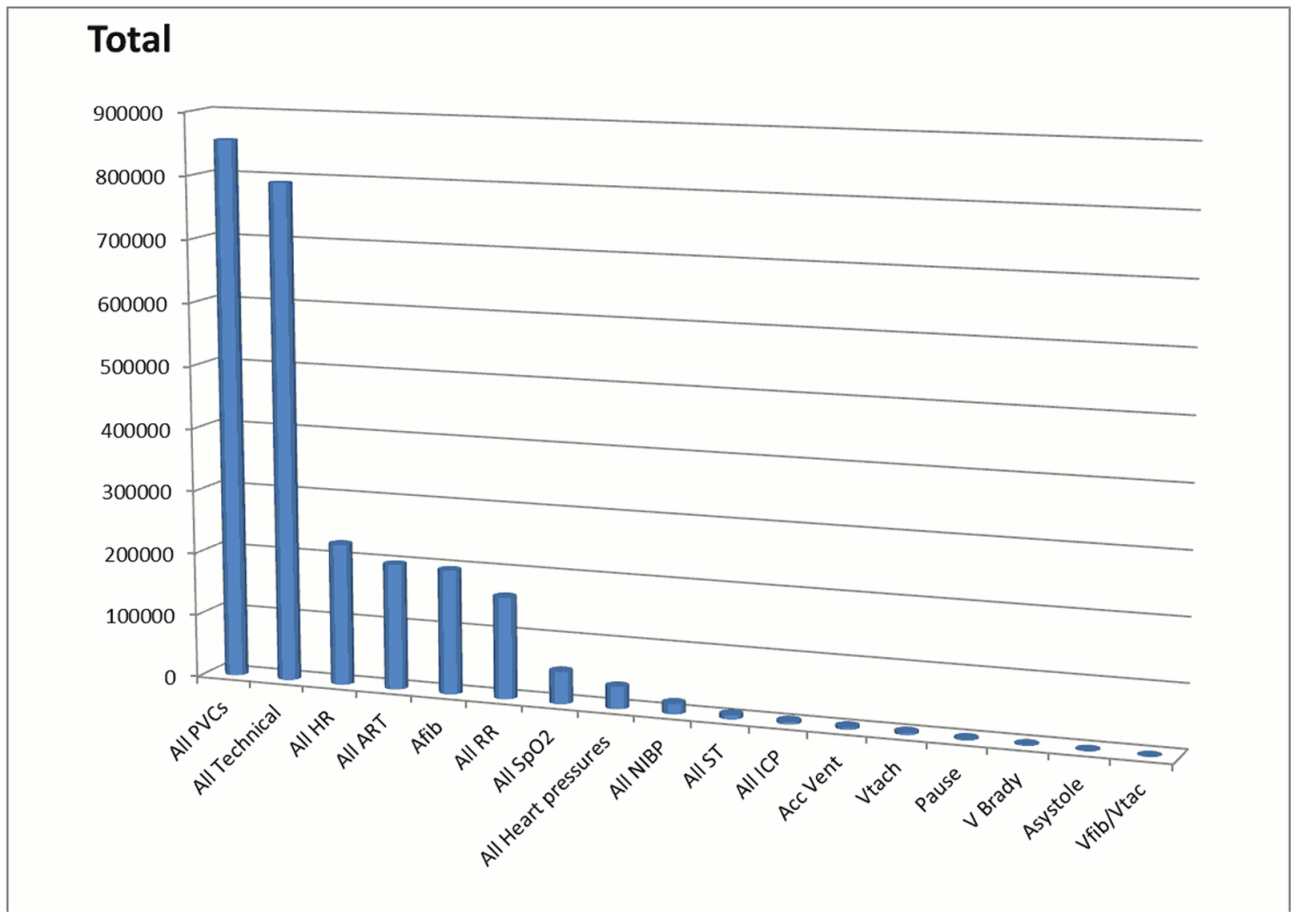
Frequency of all unique alarms (N = 2,558,760) over a 31-day period.

When looking at all audible and inaudible alarm categories (Figure 8), the alarm group that out-numbered all other alarm categories was premature ventricular contractions (PVCs) with a total of 854,901 PVC alarms during the 31-day period. The next most prevalent alarm group was technical alarms. A majority (79.4%) of the technical alarms were inaudible text message alarms (Artifact. 538,277; single Lead Fail, 90,547). Other frequent alarms were vital sign parameter threshold violations with the four most prevalent being: heart rate, invasive arterial blood pressure, respiratory rate/apnea, and SpO_2_.

Next to PVCs, atrial fibrillation was the second most frequently-occurring arrhythmia alarm. Repetitive atrial fibrillation alarms occurred in patients with persistent atrial fibrillation. For example, one patient with persistent atrial fibrillation generated 15,296 atrial fibrillation alarms and an additional 15,433 high heart rate alarms. The reason for the numerous high heart rate alarms was that this patient’s ventricular rate in atrial fibrillation averaged 130–135 which exceeded the hospital default high heart rate alarm threshold of 130. In this patient, the nurse did not tailor the alarm settings to reduce alarm burden. For example, the atrial fibrillation alarm could have been changed from an Advisory alarm to an inaudible Message alarm; the high heart rate threshold could have been increased to 150. The result of not tailoring alarm parameters to this individual patient was an average of 211 alarms per hour over the patient’s 6-day ICU stay.

### Frequency of “Treatable” Ventricular Arrhythmia Alarms

The practice guideline for treatment of patients with ventricular arrhythmias states that neither accelerated ventricular rhythm nor non-sustained ventricular tachycardia lasting less than 30 seconds warrant antiarrhythmic therapy in the hospital setting [5]. A total of 4,361 accelerated ventricular alarms occurred in the 31-day study period, all of which sounded an audible alarm because the hospital default setting was configured to be audible (Warning) for this alarm. Since no treatment is indicated for accelerated ventricular rhythm, all these audible alarms could be considered “nuisance” alarms because they are not “actionable.”

To answer the question about how many ventricular tachycardia alarms were actionable, we determined how many true ventricular tachycardia alarm events lasted 30 seconds or longer. There were a total of 502 ventricular tachycardia alarms that were determined to be true positives. Of these, 334 occurred in one patient with “ventricular storm” whose implantable device readily terminated each arrhythmia event. Excluding this one patient with device-terminated ventricular tachycardia, there were 168 true positive ventricular tachycardia alarms. Of these, 25 (14.9%) were sustained for 10 seconds or longer and 12 (7.1%) were sustained for 30 seconds or longer. All 12 of these alarms that persisted for 30 seconds or longer were life-threatening events that occurred in 6 patients. Three patients had “do not resuscitate” orders and all 3 died; the remaining 3 patients had full resuscitation attempts with 2 patients dying and one who survived to hospital discharge.

### Frequency of Respiratory Rate (RR) and Apnea Alarms

**Figure 9:**
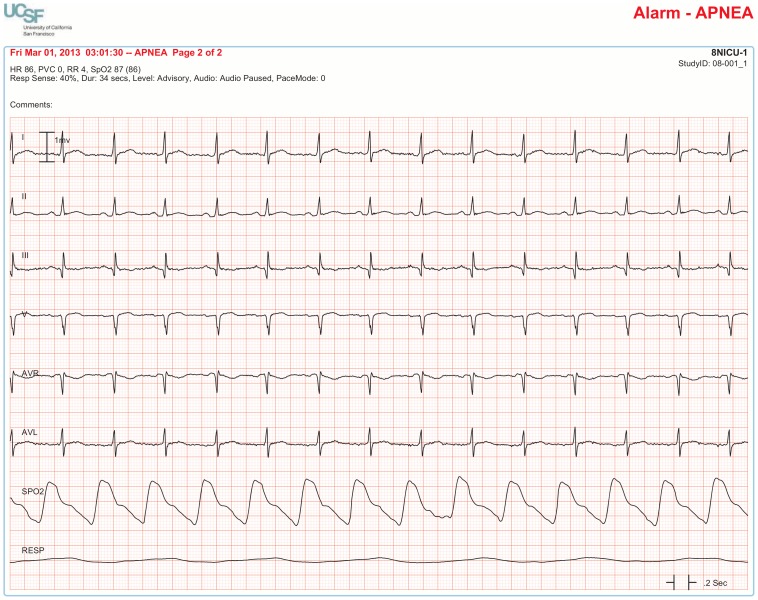
False apnea alarm in a patient breathing adequately on mechanical ventilation. The respiratory waveform (bottom tracing labelled “Resp”) has a flat line appearance. The detection of respirations from the ECG lead (impedance method) is inaccurate in this patient, displaying an erroneous respiratory rate of 4 per minute.

There was a total of 161,931 apnea or respiratory rate parameter alarms in the five ICUs over the 31-day period (average, 79 alarms/bed/day). Although apnea alarms were not annotated by the investigators, the respiratory waveform observed during arrhythmia alarm annotation often had a flat line appearance (Figure 9) in patients who were known to be breathing adequately (i.e., no respiratory arrest/need for intubation or breathing with mechanical ventilation).

### Frequency of ST-segment Monitoring Alarms

The overall frequency of ST alarms was less because only one of the five ICUs uses the ST-segment monitoring feature. In that 16-bed cardiac ICU, there was an average of 200 ST alarms per day despite a wide alarm threshold setting requiring a change in ST amplitude of ±2 millimeters for triggering an alarm. The ECG criterion for transient myocardial ischemia is a 1 millimeter ST amplitude change lasting for at least one minute [6]. As shown in Table 5, only a small proportion (9%) of the total 6,196 ST alarm events persisted for more than one minute; 91% could be considered non-actionable or nuisance alarms.

**Table 5:** ST-Segment Alarm Durations in a 16-Bed Cardiac ICU.

Alarm Duration (Seconds)	Number of Alarm Events	Percentage
0<30	4,981	80%
30<60	673	11%
>60	542	9%
Total:	6,196	

### Alarm Accuracy

Six arrhythmia alarms that were audible according to the adult ICU hospital default settings were annotated including asystole, ventricular fibrillation, ventricular tachycardia, accelerated ventricular rhythm, pause, and ventricular bradycardia. The accuracy of these alarms is shown in Table 6. Of the 461 patients, 250 patients generated at least one of the six alarm types for a total of 12,671 alarms for annotation. Only 15 (0.11%) alarms could not be determined as true versus false positives by the investigators. Although an invasive arterial pressure waveform was present in 28% and a SpO_2_ waveform was present in 96% of the alarm annotations, very few alarms required non-ECG waveforms to diagnose. In fact, 11,852 of the total 12,671 alarms (94%) could be diagnosed from analysis of the seven available ECG leads.

**Table 6:** Accuracy of 12,671 Arrhythmia Alarms.

Alarm Type	Number ofAlarms	Number ofPatients	Number of TruePositives	Number of FalsePositives	False PositiveRate
1. Asystole	792	113	260	531	67.0%
2. Ventricular Fibrillation	158	19	107	51	32.3%
3. Ventricular Tachycardia	3861	183	502	3352	86.8%
4. Accelerated Ventricular Rhythm	4361	99	224	4135	94.8%
5. Pause	2239	140	272	1963	87.7%
6. Ventricular Bradycardia	1260	39	40	1219	96.7%
**TOTAL**	12671*		1405	11251	88.8%

*15 alarms were indistinguishable: 1 Asystole, 7 VTach, 2 AccVent, 4 Pause, and 1 Ventricular Brady.

#### Accuracy of Ventricular Tachycardia and Ventricular Fibrillation Alarms

**Figure 10:**
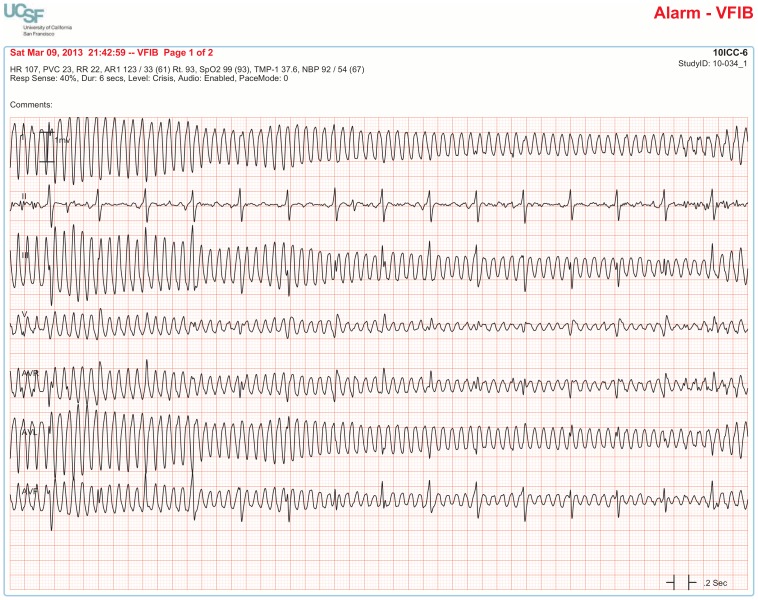
False alarm with one non-artifact ECG lead that confirms artifact mimicking ventricular fibrillation. Six of the seven ECG leads show what looks like a rapid (>400) polymorphic ventricular arrhythmia. However, Lead II clearly shows sinus rhythm at a rate of 94. Without this single non-artifact lead, a misdiagnosis would be made of ventricular fibrillation.

What was critical for the diagnosis of ventricular tachy-arrhythmia alarms was the visualization of all seven ECG leads because, not uncommonly, artifact mimicking a ventricular arrhythmia contaminated all but one lead and without observing this non-artifact lead, a misdiagnosis would have been likely (see Figure 6). Of the 51 cases of false positive ventricular fibrillation alarms, 8 (16%) had a single non-artifact ECG lead visible to identify artifact mimicking ventricular fibrillation (Figure 10). Of importance, the single non-artifact lead was often not being displayed at the bedside or central station monitors so a misdiagnosis was likely unless the clinician took the time to print out the alarm event in all seven ECG leads.

#### Accuracy of Accelerated Ventricular Rhythm Alarms

**Figure 11:**
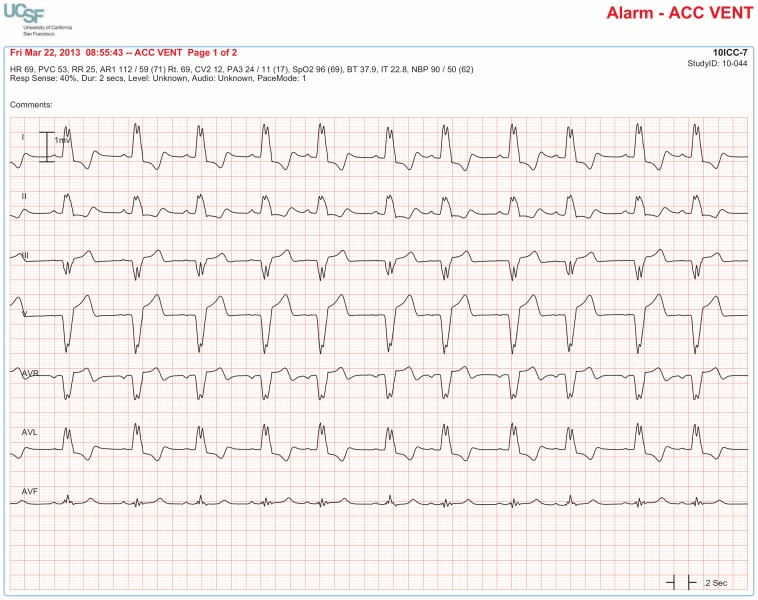
False accelerated ventricular rhythm alarm in a patient with left bundle branch block. Sinus rhythm at a rate in the 60′s is evident by observing P waves preceding each QRS complex with a consistent PR interval. P waves are visible in all seven leads (especially clear-cut in Leads I and II).

**Figure 12:**
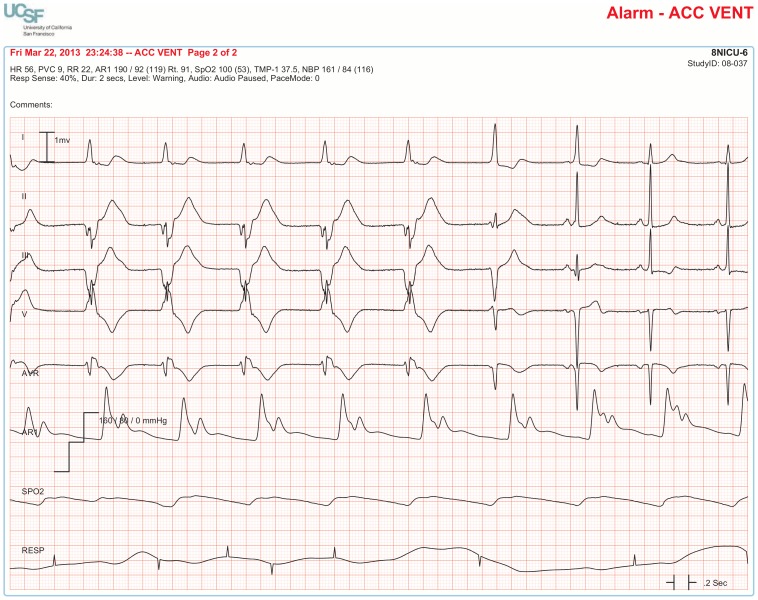
True accelerated ventricular rhythm alarm showing why this arrhythmia is not considered an “actionable” alarm condition. Accelerated ventricular rhythm at a rate of 56 for the first 5 beats followed by 2 fusion beats; the last 2 beats are normal sinus rhythm. The invasive arterial pressure waveform (bottom tracing) shows no change between normal rhythm and accelerated ventricular rhythm which confirms the rationale for the published guidelines stating no treatment is indicated for this arrhythmia in hospital settings [5].

Accelerated ventricular rhythm alarms were false in 94.8% of the 4,361 cases. A common finding in these false alarm cases was that the patient had a wide QRS complex due to a pre-existing right or left bundle branch block (Figure 11). In annotating the 4,361 accelerated ventricular rhythm alarms, we often observed in patients with concomitant invasive arterial pressure waveforms that this rhythm was not hemodynamically significant (Figure 12). Moreover, not a single accelerated ventricular rhythm alarm was associated with a Code Blue event.

**Figure 13:**
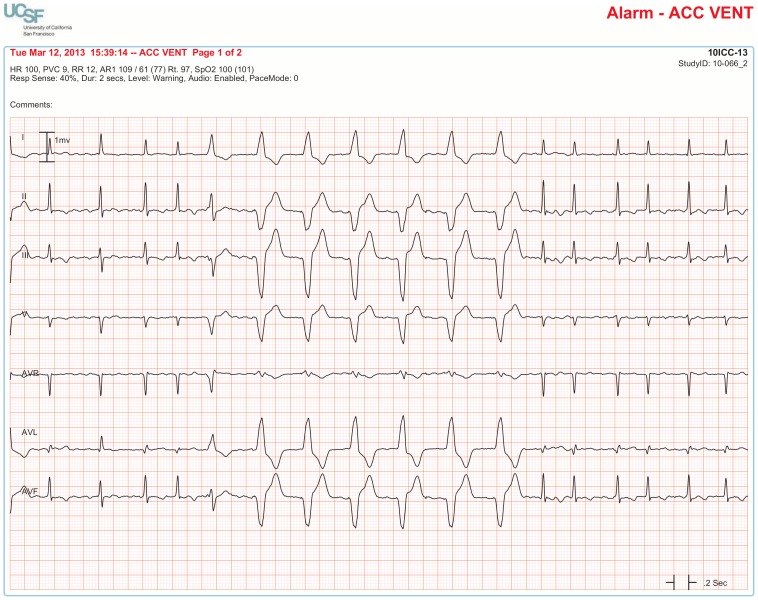
False accelerated ventricular rhythm alarm in a patient with ventricular pacing. Patient with atrial fibrillation and intermittent ventricular pacing does not have PaceMode activated. As a result, a period of ventricular pacing goes undetected by the algorithm (no pacemaker spikes are “painted” in) and a false alarm is generated of accelerated ventricular rhythm. The investigators determined this to be intermittent pacing (rather than accelerated ventricular rhythm) because the rate matched the pacemaker heart rate setting. Moreover, the QRS morphology across all 7 leads matched the QRS morphology of corresponding leads on a hospital-acquired standard “diagnostic” 12-lead ECG during a known period of pacing.

Another cause of false accelerated ventricular rhythm alarms was intermittent ventricular pacemaker rhythm (Figure 13). The monitor manufacturer requires the user to activate a feature called “PaceMode” for all patients with ventricular pacemakers. Activation of the PaceMode feature changes the frequency setting the algorithm uses to detect high frequency pacemaker stimuli (pacer “spikes”). When pacemaker stimuli are sensed by the arrhythmia algorithm, an artificial spike is “painted” in for clinicians to readily identify pacing rhythm. We found that only 33.3% of patients with pacemakers had the PaceMode feature activated.

#### Accuracy of Brady-Arrhythmia Alarms

**Figure 14:**
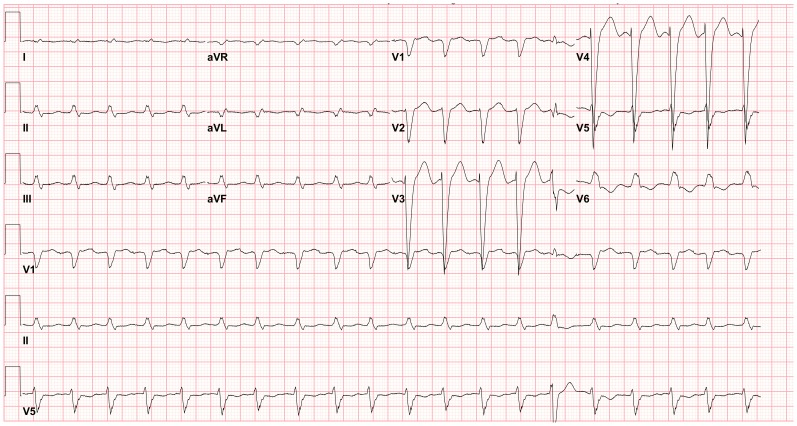
Low amplitude QRS in a patient with an excessive number of alarms. Standard “diagnostic” 12-lead ECG recorded from the patient who contributed nearly half of the 12,671 arrhythmia alarms for annotation. The ECG shows left bundle branch block with low amplitude QRS complexes in the limb leads but not in the V leads. Since one of the available leads acquired with the physiologic patient monitoring device is a V lead, the arrhythmia algorithm could have avoided the excessive number of false alarms had all available leads been used for QRS detection.

A serendipitous discovery during arrhythmia alarm annotation was that patients who had low amplitude QRS complexes, especially in the limb leads, had a lot of false brady-arrhythmia alarms (asystole, pause, ventricular bradycardia). Figure 14 shows the hospital-acquired standard “diagnostic” 12-lead ECG of the patient who contributed the most alarms for annotation. This one patient contributed 5,725 of the 12,671 arrhythmia alarms for annotation (45.2%). Although this patient’s QRS amplitude was low in the six limb leads, the V lead had adequate amplitude for an algorithm to sense. Therefore, if the arrhythmia algorithm had used all available leads to identify QRS complexes, the problem of under-counting heart rate and false brady-arrhythmia alarms could have been avoided.

Table 7 shows an analysis of how many asystole or pause false alarms had visible QRS complexes in at least one of the available ECG leads. The vast majority of these false alarms (91% of false asystole alarms; 94% of false pause alarms) had visible QRS complexes in one or more leads that could have been detected by the arrhythmia algorithm had all available leads been used.

**Table 7:** Frequency of Visible QRS Complexes in One or More ECG Leads during False Brady-Arrhythmia Alarms.

Alarm Type	Visible QRS Complex	
	Yes	No
False Asystole Alarms, N = 518	469 (91%)	49 (9%)
False Pause Alarms, N = 1903	1786 (94%)	117 (6%)

### Effect of Signal Quality on False Arrhythmia Alarm Rate

**Figure 15:**
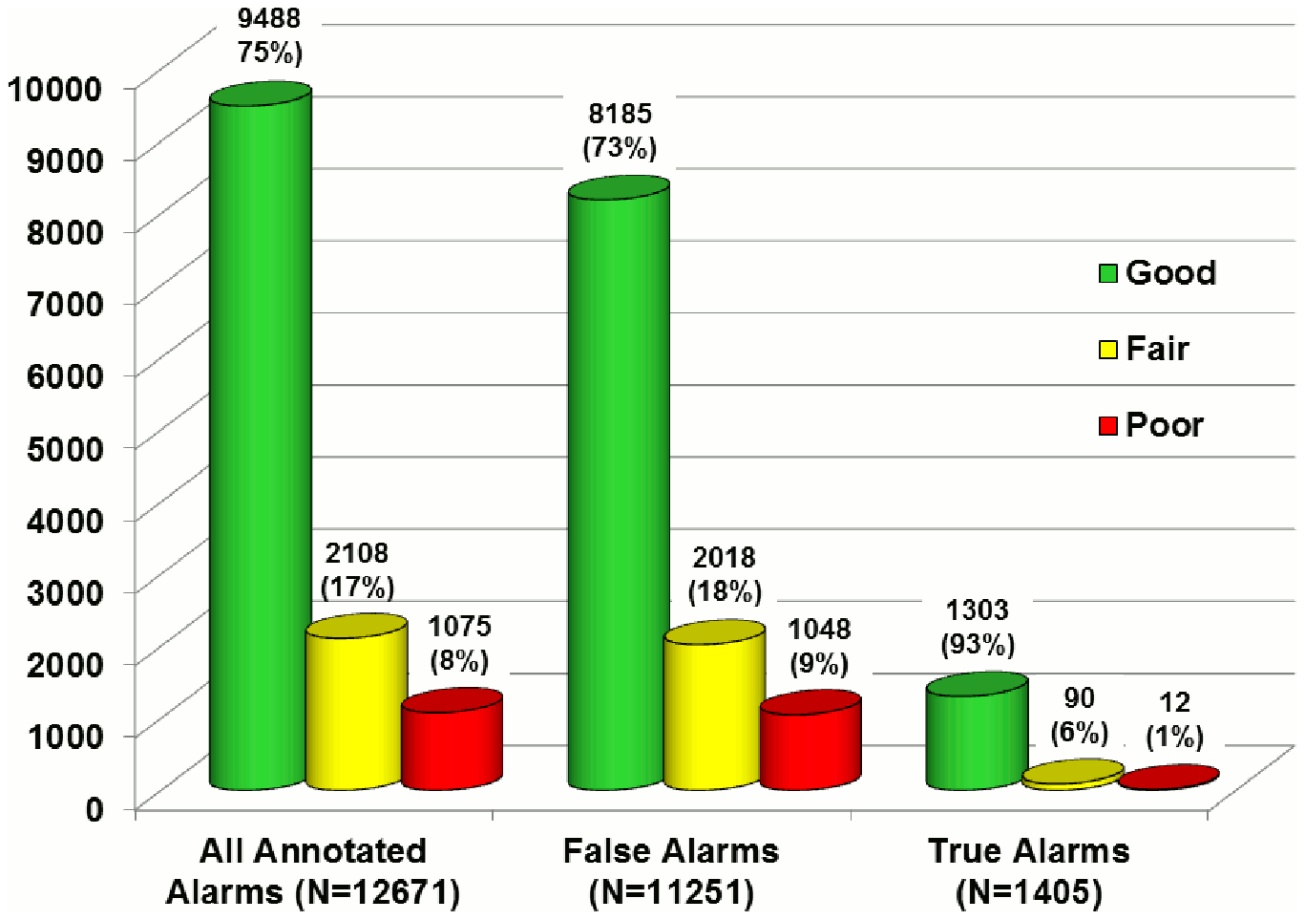
ECG signal quality in 12,671 annotated arrhythmia alarms. Good signal quality (green) was defined as a clearly visible P-QRS-T waveform across all available leads with little to no noise, baseline wander, or leads off. Fair signal quality (yellow) was defined as moderate noise or baseline wander but having identifiable QRS complexes for basic rhythm/rate detection. Poor signal quality (red) was defined as being unanalyzable because of excessive noise, baseline wander or leads off.

**Figure 16:**
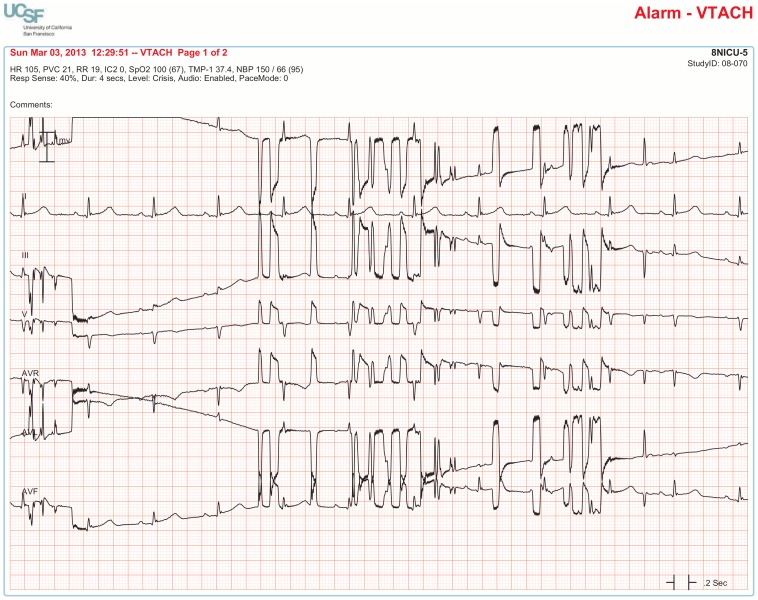
Electrode failure causing artifact and a false ventricular tachycardia alarm. Electrocardiogram in 6 of the 7 available leads shows intermittent loss of signal (signal “squares off” on top and bottom of tracing) due to an electrode problem such as loss of skin contact or dried out electrode gel. One ECG lead (Lead II that uses the right arm and left leg electrodes) does not show electrode failure so the likely electrode that is malfunctioning is the left arm electrode. Failure to apply fresh electrodes in this case will result in numerous false alarms.

A majority (74.9%) of the annotated arrhythmia alarms were rated by the investigators as having good signal quality at the time the alarm was triggered (Figure 15). Only 8.5% of alarms had poor signal quality. False alarms had a higher proportion of poor signal quality (9.3%) compared with true alarms (0.9%); however, our findings indicate that poor signal quality was not a major cause of the excessive number of arrhythmia alarms. In rare instances, there was clear-cut evidence of an electrode problem triggering a false alarm as shown in Figure 16.

## Discussion

The present study represents the largest (N = 12,671) annotated arrhythmia alarm database to date from all consecutive patients treated in full service hospital adult ICUs. The only comparable database is the arrhythmia alarm annotation subset from the Multiparameter Intelligent Monitoring in Intensive Care (MIMIC II) database [7] (see Table 8 for a comparison). The MIMCI II investigators reported an alarm burden of 48 alarms per bed per day [8] which is lower than our audible alarm burden of 187 alarms per bed per day. A likely explanation for this difference is that the alarms annotated in the MIMIC II database included only patients who had invasive arterial pressure monitoring. This selects for a more sedentary sample because patients with arterial pressure monitoring are sedated so they don’t inadvertently disconnect their arterial line and hemorrhage to death. Therefore, the MIMIC II ECG waveforms would not be expected to contain as much motion artifact triggering alarms as in our “real world” database.

**Table 8:** Comparison of Annotated Arrhythmia Alarm ICU Databases.

Database Characteristics	MIMIC II*	UCSF
Monitor manufacturer	Philips Healthcare	GE Healthcare
Number, type of patients	N = 447; had to have both ECG & invasive arterial pressure	N = 461; all consecutive patients
Number, type of beds	48 beds; medical, surgical, cardiac	77 beds; medical, surgical, cardiac, neurologic
Total monitoring hours	41,301 hours	48,173 hours
Number, type of ECG leads	1–3 leads; inconsistent, including modified (MCL) leads	7 leads; consistently I, II, III, aVR, aVL, aVF, V_1_
Sampling rate	125 HZ	ECG, 240 HZ; Pressures, 120 HZ; SpO_2_, 60 HZ
Resolution	8 bit	12 bit
Number of alarms annotated	5,386 alarms; 5 alarm categories	12,671 alarms; 6 alarm categories
Asystole	579	792
Vfib/Vtach	313	158
Vtach	1900	3861
	Brady (<40), 717	Vbrady (≤50), 1260
	Tachy (>140), 1877	Accelerated ventricular rhythm, 4361
		Pause, 2239

*Annotation subset of the MIMIC II database [7]. Vfib = ventricular fibrillation; Vtach = ventricular tachycardia; Brady = bradycardia; Tachy = tachycardia; VBrady = ventricular bradycardia.

We found a staggering total number of alarms (>2,500,000 in one month) when counting all audible and inaudible arrhythmia, parameter, and technical alarms. Although many of these alarms were configured to be inaudible text messages, we still found a high audible alarm burden of 187 audible alarms per bed per day. A noisy alarm environment interrupts patients’ sleep and invokes fear in patients and their families. In our institution, the question on our patient satisfaction questionnaire that consistently has a low score is the one related to hospital noise.

The most prevalent group of alarms was PVC alarms that were triggered nearly 900,000 times over the one-month period. When hospital monitoring began in the late 1960’s, PVC’s were thought to lead to ventricular tachycardia or fibrillation. As a result, standing orders were established in Coronary Care Units for nurses to initiate intravenous antiarrhythmic therapy for conditions such as 5 or more PVC’s per minute, a ventricular couplet, a single PVC with “R on T” phenomenon, multiform PVC’s, ventricular bigeminy or trigeminy, or 3–5 consecutive PVC’s. Therefore, PVC alarm algorithms were developed to identify patients for whom treatment was recommended. However, treatment of PVC’s changed when a landmark clinical trial, the Cardiac Arrhythmia Suppression Trial (CAST) was published in 1989 [9]. The CAST study demonstrated that antiarrhythmic therapy was associated with more deaths than placebo. Thus, in the 25 years since the CAST report, PVCs have been considered a non-actionable alarm.

In our institution, PVC alarms are configured to be inaudible. However, there is a concern about missing patients who are at risk for the potentially lethal arrhythmia, torsade de pointes. In patients who develop a prolonged QT interval due to the initiation of a potentially proarrhythmic drug, the onset of PVC’s and ventricular couplets may be a sign of impending torsade de pointes [10]. For this reason, a smart alarm that would couple continuous QT_C_ measurement with the development of PVC’s would be beneficial to warn clinicians when a QT-prolonging drug should be discontinued to prevent torsade de pointes.

Consecutive PVCs (non-sustained ventricular tachycardia) is an arrhythmia that is often of little consequence to the patient if the rate is not excessively rapid (<140) or its duration is brief. Published practice guidelines state that only symptomatic and sustained (≥30 seconds) ventricular tachycardia should be treated in the hospital setting [5]. We found that of 168 true instances of ventricular tachycardia, only 25 (14.9%) were sustained for 10 seconds or more and only 12 (7.1%) lasted 30 seconds or more. However, all 12 of the 30-second episodes were cardiac arrest events so an arrhythmia algorithm that required the patient to be in ventricular tachycardia for ≥30 seconds before triggering an alarm would be too extreme. At our institution, the Director of the Cardiac Electrophysiology Service wants non-sustained ventricular tachycardia lasting ≥10 seconds to trigger an alarm and to be documented in the medical record whether or not the arrhythmia is treated (personal communication, Edward P. Gerstenfeld, MD on June 13, 2014). As a result of our findings and in accordance with the practice guideline that states that in-hospital treatment of non-sustained ventricular tachycardia is unwarranted, our institution has changed the hospital default setting to make VT >2 alarms (3–5 consecutive PVC’s) inaudible text messages.

It would be helpful if device manufacturers made ventricular tachycardia alarms configurable. The result of configuring ventricular tachycardia alarms to those lasting ≥10 seconds in the present study would have been a reduction of ventricular tachycardia true alarms by 85%. Moreover, the reduced number of these alarms would have been considered clinically actionable for either documentation in the medical record or antiarrhythmic treatment. In addition, smarter alarms should be developed and tested to identify which ventricular tachycardia events are likely to be symptomatic (e.g., those with rates >140 that decrease arterial pressure).

The second most prevalent alarm category was technical alarms that is in agreement with the study by Siebig and colleagues [11] who reported technical alarms to be their second most common type of alarm. It is important to point out that nearly 80% of our technical alarms were inaudible text message alarms (Artifact and single Lead Fail alarms). Inaudible text message alarms should not be considered totally innocent in causing alarm fatigue because they often alert about a problem the nurse doesn’t know how to solve which may cause anxiety. For example, if the bedside monitor displays the message “Lead I Fail”, few nurses know that they need to check the integrity of two electrodes used to make Lead I: the right and left arm electrodes. In contrast, if the monitor displays the message “Lead V Fail”, the integrity of all five electrodes should be checked. What would be more helpful is an algorithm that would monitor each electrode’s impedance and provide a message to check a specific electrode (RA, LA, RL, LL, or V).

It is unclear whether the high number of technical alarms we observed means we have a problem with electrode integrity and signal quality in our ICUs. No other study has reported on the prevalence of technical (especially inaudible message) alarms so we have no source for comparison. Most of our technical alarms (79.4%) were inaudible Artifact or single Lead Fail alarms that may be harbingers of more serious audible alarms or subsequent complete arrhythmia suspension.

It has been suggested that alarm burden could be reduced if nurses applied fresh electrodes every 24 hours [12]. It would be interesting to determine whether preventive action (electrode change) taken during the text message phase of technical alarms would reduce subsequent audible alarms and arrhythmia suspension. However, Artifact or single Lead Fail alarms could also be caused by motion artifact in a patient with intact electrodes. A brief period of motion artifact in an otherwise good signal quality ECG appeared to be the cause of many of our false arrhythmia alarms (see Figures 6 and 7). Obviously, an optimal electrode product, skin prep, and daily change regimen will not impact alarm fatigue if the source of technical and false arrhythmia alarms are due to normal patient movement.

A surprising finding was that poor signal quality was not a major cause of false arrhythmia alarms. For example, only 9% of the 11,251 false arrhythmia alarms were rated by the investigators as having poor signal quality.

Our findings confirm that nurses do not always tailor alarm settings appropriately for their individual patient. We found that hospital default settings were often left in place regardless of whether it made sense for the individual patient. While it is tempting to blame the user for alarm fatigue, device manufacturers also have a responsibility to make monitors more helpful, interactive, and intuitive. Moreover, it would be more efficient and reliable to have monitor devices suggest alarm setting changes rather than to rely on humans, who are distracted by multiple competing priorities, to remember to tailor alarms to their individual patient.

A major opportunity exists for monitor device manufacturers to track frequent repetitive alarms and to remind nurses to consider changing alarm settings. For example, if the hospital default setting for high heart rate is 130 but the patient’s rate averages 135 because of persistent atrial fibrillation, a prompt could pop up saying, *“Mean HR 135; do you want to increase high HR setting?”* A drop-down menu of rates beginning at 136 could then be provided to quickly change the threshold and reduce subsequent alarms. Then, when treatment of rapid ventricular rate is successful in lowering this patient’s rate, another prompt could say, *“Mean HR 90; do you want to restore hospital default setting of 130?”.*

Burgess, et al. examined heart rate (HR) and respiratory rate (RR) settings in 317 patients who had no adverse hospital events to determine ideal settings to minimize nuisance alarms [13]. They reported that a high HR of 130–135, low HR of 40–45, high RR of 30–35, and low RR of 7–8 were optimal for this stable cohort. While such analyses may provide guidance for hospital default settings, a “one size fits all” approach is inappropriate because of large variations between individual patient’s vital sign measurements. We believe parameter threshold settings should be tailored to the individual patient for maximal reduction of nuisance alarms.

In clinical practice, it is important to detect when patients develop atrial fibrillation so the nurse can assess how the patient is tolerating the arrhythmia and prompt treatment can be initiated to slow the ventricular rate and restore sinus rhythm. Likewise, it is important to detect when atrial fibrillation terminates because patients should be assessed for embolic events (stroke, peripheral arterial emboli) at the time of cardioversion. Thus, a monitor alarm at atrial fibrillation onset and termination would be clinically useful. However, it is not necessary to have repetitive atrial fibrillation alarms for patients with persistent atrial fibrillation when treatment has already been initiated or for patients with permanent (chronic) atrial fibrillation where the goal is not to terminate the arrhythmia. In such cases, a prompt could pop up saying, “*Do you want to continue to hear atrial fibrillation alarms?”* If the nurse replied “no”, then the alarm could automatically be switched to an inaudible text message.

A feature the device manufacturers should provide to help clinicians diagnose arrhythmia alarms more accurately is to make it easy (“one step”) to visualize and print out all available ECG leads at the time an arrhythmia alarm is triggered. We found that we could diagnose whether an arrhythmia alarm was true or false in 94% of the 12,671 cases by observing the seven available ECG leads; we rarely needed additional non-ECG waveforms (e.g., arterial pressure or SpO_2_ waveforms) to reach a diagnosis. Not uncommonly, false ventricular arrhythmia alarms were triggered by artifact that contaminated all but one ECG lead and this lead was often not a lead being displayed at the bedside or central station.

Likewise, if the arrhythmia algorithm took advantage of all available leads, it is likely that the high false alarm rate could be significantly reduced. If only one alarm in 10 is a true arrhythmia, it is not surprising that this “cry wolf” phenomenon results in staff ignoring alarms. If, however, the arrhythmia algorithm could identify a non-artifact lead for analysis and reduce the false alarm rate, clinicians would rapidly perceive that alarms were clinically meaningful and respond accordingly.

An additional condition triggering false alarms that could be mitigated by an analysis of all available ECG leads is the problem of low QRS amplitude. Low amplitude QRS complexes can occur in the morbidly obese, patients with pericardial effusions, and altered conduction such as bundle branch block. The outlier patient in the present study who generated more arrhythmia alarms for annotation than any other patient (45% of the total 12,671 annotated alarms) had low amplitude QRS complexes in the limb, but not precordial leads due to left bundle branch block.

The American National Standard for cardiac monitors, heart rate meters and alarms [14] states that the device should not detect a QRS if the waveform is less than 0.15 mV (1.5 millimeters) in size. This standard was designed to prevent the monitor from misdiagnosing P waves as QRS complexes during ventricular standstill. However, monitor manufacturers elect to use a higher QRS detection threshold (e.g., 0.5 mV) and they also may require that this higher threshold be present in more than one ECG lead. In addition, the device may measure only the portion of the QRS complex that points in one direction rather than measuring from “peak to trough” as the National Standard recommends. This results in undercounting of amplitude in biphasic QRS complexes. All of these measurement decisions result in heart rate undercounting and false asystole, pause, and bradycardia alarms.

Failure to detect low amplitude QRS complexes is probably a systemic problem with monitor manufacturers. For example, investigators in the MIMIC II trial (Philips monitor devices) reported an even higher false alarm rate for asystole (90.7%) [7] than our current findings with GE monitor devices (false asystole alarms, 67%).

We found that when we observed all seven available ECG leads, a sizable QRS complex was readily visible in one or more lead in 91% of the false asystole alarms and in 94% of the false pause alarms. An alternative approach that would reduce false alarms would be to count waveforms as small as 0.15 mV from peak to trough as QRS complexes, especially if they match the pulsatile rate of SpO_2_ or arterial pressure waveforms.

One arrhythmia alarm that generated a lot of false alarms (95% false) but did not cause a drop in arterial pressure or cardiac arrest was accelerated ventricular rhythm. It is not surprising that a ventricular rhythm at a rate of 50–100 would be well tolerated by the patient because this arrhythmia is analogous to ventricular pacing in this rate range. As a result of our findings and in accordance with the practice guideline that states that in-hospital treatment of accelerated ventricular rhythm is unwarranted [5], our institution has changed the hospital default setting to make accelerated ventricular rhythm alarms inaudible text messages. This change alone is anticipated to eliminate more than 4,000 audible alarms per month in our adult ICU’s.

Whalen, et al. took a completely different approach to accelerated ventricular rhythm alarms in their Quality Improvement Project by configuring them to the highest level of crisis alarms [15]. Their rationale was to “ensure that nursing staff viewed these alarms as they occurred.” ([15], page 4) However, it is unclear why staff should be bothered by a noxious audible alarm for an arrhythmia event that is well tolerated by the patient and does not warrant treatment according to published practice guidelines [5].

Patients with intermittent ventricular pacing often trigger false ventricular alarms, especially when the nurse has neglected to activate the PaceMode feature that the device requires to detect high frequency pacemaker stimuli (pacer “spikes”). We found that only one third of patients who had pacemakers had the PaceMode feature activated. This provides another opportunity for the device manufacturer to search for pacer spikes in all patients so that we are not dependent upon humans to remember to activate this feature.

Although respiratory waveform annotation was not performed in our study, anecdotal evidence of flat-line respiratory waveforms in patients we knew were on mechanical ventilation or breathing normally suggests a problem with detection of respiration using impedance methods. Manual searching by the user for the best respiratory ECG lead is a time-consuming and often neglected task. Thus, it would be beneficial for the monitor device to help the user by automatically searching for the best available ECG lead for a respiratory waveform of adequate size for detection. An area for future research is to develop and test other, and perhaps more accurate, measures of respiratory rate and apnea detection such as those derived from subtle QRS morphology changes with inspiration and expiration [16].

Two parameter alarms that lend themselves to adding delays before an alarm is triggered are SpO2 and ST-segment alarms. For both of these parameters, brief spikes in the trend are not indicative of a pathophysiologic process. Welch [17] reported that increasing alarm delays from 5 to 15 seconds could decrease alarms by 70%. However, this was an off-line retrospective analysis with no data collected on what effect this alarm delay might have on patient outcomes. We are currently completing a prospective, randomized clinical trial to test whether the addition of a SpO_2_ alarm delay of 20 seconds plus a change of SpO_2_ default threshold from 90% to 88% will reduce SpO_2_ alarm burden without causing adverse patient outcomes.

In terms of ST-segment monitor alarms, we found that the vast majority (91%) of these alarms represented brief spikes of ST amplitude change rather than the gradual ST change that is indicative of transient myocardial ischemia. We previously reported that the most frequent cause of false ST-segment monitor alarms is a body position change that typically causes a quick change in ST amplitude [18]. Therefore, ST alarms should be configurable so a one-minute delay could be set for ST segment changes in accordance with the Holter guideline criteria for the diagnosis of transient myocardial ischemia [6]. In addition, acute myocardial ischemia produces ST changes in at least two contiguous ECG leads [19]. As a result, requiring the ST change to last at least one minute and to be present in two contiguous leads would drastically reduce the number of false ST-segment monitor alarms. Whereas contiguous precordial leads are generally not monitored in hospital settings, all six limb leads are routinely available. Contiguity in the limb leads should be defined as the following sequence: aVL, I, minus aVR, II, aVF, III [20].

Table 9 provides a summary of the conditions observed in the present study that cause excessive physiologic monitor alarms. The table also includes suggestions for device solutions.

**Table 9:** Key Insights into the Problem of Alarm Fatigue and Recommendations.

Conditions Causing Excessive Alarms	Suggestions for Device Improvements
1. Alarms are not tailored to the individual patient	• Have monitor prompt more appropriate alarm settings; e.g., *“Mean HR = 130; do you want to increase high HR setting?”*
2. Persistent atrial fibrillation	• Have monitor trigger alarms only upon new onset or termination of atrial fibrillation
	• When there are repetitive audible alarms, have monitor prompt *“Do you want to continue to hear Afib alarms?”*
3. Artifact mimics VT or VFib	• Have arrhythmia algorithm use all available ECG leads to identify a non-artifact lead
	• Make it easy to view and print out all available ECG leads at the time the alarm was triggered
4. Low amplitude QRS causes pause, asystole, & bradycardia false alarms	• Have arrhythmia algorithm use all available ECG leads to identify QRS complexes
	• Detect QRS if ≥1 lead has peak-to-trough amplitude of ≥0.15 mV as allowed by the AAMI standard, especially if rate matches SpO_2_ or arterial pressure waveforms
5. Wide QRS due to BBB or pacemaker rhythm triggers ventricular arrhythmia alarms	• Have monitor detect high frequency pacemaker “spikes” without clinician having to tell the monitor the patient has a pacemaker
	• Have monitor algorithm identify P waves to distinguish sinus rhythm with BBB
6. VT alarms not “actionable”	• Make VT alarm delays configurable according to criteria for documentation (≥10 seconds) or treatment (30 seconds) established by hospital preferences & practice guidelines
	• Use VT rate, invasive arterial pressure and SpO_2_ to identify hemodynamically significant (symptomatic) VT
7. Electrode failure causes poor signal quality	• Have monitor measure each electrode’s impedance and indicate when one is failing so electrode can be changed
8. ST-segment alarms are not truly indicative of myocardial ischemia	• Make ST alarm delays configurable according to criteria for ischemia (lasting 1 minute) to prevent brief “spikes” in ST amplitude from triggering alarms
	• Define ischemia only when present in 2 contiguous limb leads in order of aVL, I, minus aVR, II, aVF, III)
9. Flat-line respiratory wave-form cause false apnea & RR alarms	• Have monitor automatically search for best available ECG lead to measure/display respiration waveform
	• Investigate ECG-derived respiratory measurement to replace impedance method

Afib = atrial fibrillation; AAMI = Association for the Advancement of Medical Instrumentation; HR = heart rate; BBB = bundle branch block; VT = ventricular tachycardia; VFib = ventricular fibrillation; RR = respiratory rate.

### Study Limitations

The focus of the present study was to gain insight into the cause of excessive alarms, especially false alarms. We cannot report on any false negatives that may have been missed in this cohort of patients. However, we are confident that no lethal event was missed by the monitor device because all of the 17 Code Blue events were heralded with multiple arrhythmia and parameter alarms.

### Areas for future research

There is inadequate data on commercially-available electrodes and how often they should be changed to prevent false alarms due to electrode failure. It is also unknown what impedance measurement would indicate electrode failure and how best to measure impedance continuously. There is also insufficient data on the best skin prep regimens that will decrease electrode impedance without causing skin breakdown.

Investigators have reported that adding alarm delays for SpO_2_ [17] or other parameters (heart rate, respiratory rate, arterial and noninvasive blood pressure) [21] would be effective in reducing alarm fatigue. However, these studies have not measured the effect of such delays on patient outcomes. Thus, future research is required to determine whether these alarm delays are safe as well as effective in reducing alarm burden.

Any algorithm change to incorporate recommendations in Table 9 should be tested to determine the effect on false alarm rates and identification of any unintended adverse consequences. A “gold standard” database of annotated alarms should be made available for such testing. Organizations like the Association for the Advancement of Medical Instrumentation (AAMI), the U. S. Food & Drug Association’s (FDA’s) Center for Radiological Devices and Health, and the National Institutes of Health in partnership with professional societies like the International Society for Computerized Electrocardiology, the American Association of Critical-Care Nurses, and the American Heart Association could foster such a gold standard database.

In summary, the excessive number of physiologic monitor device alarms is a complex interplay of inappropriate user settings, patient conditions, and algorithm deficiencies. Because computer devices have the potential to be more reliable than humans, an opportunity exists to improve physiologic monitor devices to reduce the problem of clinical alarm fatigue.
